# Neurological soft signs and cognition in the late course of chronic schizophrenia: a longitudinal study

**DOI:** 10.1007/s00406-020-01138-7

**Published:** 2020-05-16

**Authors:** Christina J. Herold, Céline Z. Duval, Johannes Schröder

**Affiliations:** Section of Geriatric Psychiatry, Department of General Psychiatry, University of Heidelberg, Voßstr. 4, 69115 Heidelberg, Germany

**Keywords:** Neurological soft signs, NSS, Chronic schizophrenia, Follow-up, Neuropsychology, Psychopathology

## Abstract

Neurological soft signs (NSS) are minor (‘soft’) neurological abnormalities in sensory and motor performances, which are frequently reported in patients with schizophrenia at any stage of their illness. It has been demonstrated that NSS vary in the clinical course of the disorder: longitudinally NSS decrease in parallel with remission of psychopathological symptoms, an effect which mainly applies to patients with a remitting course. These findings are primarily based on patients with a first episode of the disorder, while the course of NSS in patients with chronic schizophrenia and persisting symptoms is rather unknown. Therefore, we investigated NSS twice in 21 patients with chronic schizophrenia (initial mean duration of illness: 23 ± 11 years) with a mean follow-up interval of 7 years. NSS were evaluated by the Heidelberg Scale, established instruments were used to rate neuropsychological performance and psychopathological symptoms. NSS showed significant increases on the subscales “motor coordination” and “integrative functions”, while positive and negative symptoms, including apathy, showed only minor, non-significant changes. Verbal memory, verbal fluency, and cognitive flexibility along with severity of global cognitive deficits demonstrated a significant deterioration. Regression analyses identified executive dysfunction (cognitive flexibility and verbal fluency) at baseline as significant predictors of NSS increase at follow-up. Our findings indicate that NSS deteriorate in the long-term course of chronic schizophrenia. This effect may be accounted for by a decrease of executive functions and logical memory, which can be attributed to premature brain aging.

## Introduction

Neurological soft signs (NSS) represent a variety of discrete abnormalities in sensory integration, motor coordination and sequencing of complex motor acts and are demonstrated in the majority of patients with schizophrenia, including neuroleptic-naïve first-episode patients and chronic cases [1–4].

Previous studies clearly show that NSS are not a static feature of schizophrenia, but instead vary in the clinical course of the disorder. This effect was confirmed in a meta-analysis based on 17 longitudinal studies, which yielded a reduction of NSS in the course of acute psychosis paralleling the remission of psychopathological symptoms [5]. However, NSS scores did not normalize to the levels typically found in healthy controls. Recently, Bachmann and Schröder [6] presented an overview about 29 follow-up studies, which addressed NSS longitudinally in patients with schizophrenia at different stages of their illness. While patients with a first-episode or a remitting course predominantly showed a decrease of NSS scores over time, levels of NSS increased in those with an unfavorable, chronic course. With respect to effects of medication, there seems to be a relationship between medication response and improvement of NSS scores independent of the type of antipsychotics.

Moreover, in a longitudinal MRI (magnetic resonance imaging)-study of our group, Kong et al. [7] confirmed this dichotomization, which was reflected by the underlying cerebral changes: 20 patients with first-episode schizophrenia (mean age: 25.6 ± 7.2 years) were investigated twice, after remission of the acute symptoms and after a follow-up period of one year. While patients with decreasing NSS scores showed rather minor and localized changes within the left frontal lobe, cerebellum and cingulate gyrus, patients with persistent NSS scores demonstrated pronounced gray matter (GM) reductions over time. Their more unfavorable course was associated with GM decreases of the sublobar claustrum, cingulate gyrus, cerebellum, frontal lobe and middle frontal gyrus. These findings correspond to results from other longitudinal and cross-sectional (first-episode vs. chronic schizophrenia) MRI-studies, which yielded progressive structural brain abnormalities in schizophrenia in frontal, cingulate and cerebellar regions [for review see: 8, 9–11].

While neuropsychological deficits are among the core features of schizophrenia [12–15], studies of patients with chronic schizophrenia demonstrated that their test performance further decreases [16–18]. An age-associated cognitive decline seems to be especially pronounced in some domains such as cognitive flexibility [12]. These findings were contrasted by a rather stable psychopathological profile cross-sectionally with unchanged negative symptoms and slightly elevated positive symptoms in the youngest subgroup [12, 19].

Along with risk factors such as lower educational attainment, persistent psychopathology and advanced age that may increase cognitive decline in older individuals with schizophrenia, concomitant physical diseases like hypertension, adiposity or diabetes mellitus might also contribute to the observed cognitive and functional decline in older patients with a chronic course of the disease [20–22]. However, only a few studies focused on patients with chronic schizophrenia and/or the course of the disorder during late adulthood. This issue is of particular relevance given the growing number of older patients with schizophrenia [23].

The aim of the present study was to investigate the longitudinal course of NSS in a sample of severely disabled patients with chronic schizophrenia. Based on the given literature, we expected NSS scores to further increase over time, accompanied by a deterioration of neurocognitive functions.

## Methods

### Subjects

We examined 21 patients with chronic schizophrenia (*N* = 18) or schizoaffective (*N* = 3) disorder [DSM-IV criteria, 24] twice, 2008/2009 (T1) and 2015–2017 (T2) (see Table 1). Patients were recruited from three psychiatric long-term units (T1: *N* = 20, T2: *N* = 13) and a mental state hospital (T1 and T2: *N* = 1), respectively; at T2, 7 patients had been placed in nursing homes. Patients were treated with antipsychotic medication according to their psychiatrists’ choice. Initially, all but 4 patients received exclusive atypical antipsychotics, at the follow-up evaluation 8 patients received both, atypical and typical antipsychotic medication, and one received typical antipsychotic medication only.

**Table 1:** Demographic and clinical characteristics

	T1 *n* = 21	T2 *n* = 21
	Mean (SD)	Mean (SD)
Education, years	11.67 (2.46)	
Sex, *N*, % masculine	15, 71.4%	
Duration of illness, years	23.05 (11.09)	30.71 (9.97)
Age, years	45.33 (8.71)	52.71 (8.70)
Age at illness onset, years	22.00 (8.40)	

Psychiatric history of the patients was retrieved from medical records. Given that our sample almost exclusively comprised severe disabled patients living in psychiatric-long-term units, physical comorbidities as hypertension (T2 *N* = 11), adiposity (BMI ≥ 30, T2 *N* = 10), diabetes mellitus (T2 *N* = 7), hyperlipidemia (T2 *N* = 14), nicotine abuse (T2 *N* = 15) or alcohol/drug abuse in the past (T2 *N* = 5) were frequently diagnosed. Patients with extrapyramidal side effects, akathisia, parkinsonian signs and abnormal involuntary movements were excluded before study entry, as it was the case for patients with late onset schizophrenia, i.e., with a manifestation of the disease after age 40 [16]. Due to the chronicity of illness, not all participants were able to perform all neuropsychological tasks and NSS items.^1^

The investigations were approved by the ethics committee of the Medical Faculty, Heidelberg University. After full explanation of the proceedings, subjects provided written informed consent to participate in accordance with the Declaration of Helsinki.

### Clinical and neuropsychological assessments

Psychopathological symptoms were assessed on the Brief Psychiatric Rating Scale [BPRS, 25], the Scales for the Assessment of Positive and Negative Symptoms [SAPS and SANS, 26, 27] and the Apathy Evaluation Scale [AES, 28, 29].

NSS were examined with the Heidelberg Scale [2, 30] that comprises motor and sensory NSS. The scale consists of five items assessing motor coordination,^2^ three items assessing integrative functions,^3^ two items assessing complex motor tasks,^4^ four items assessing right/left and spatial orientation^5^ and two items assessing hard signs.^6^ Ratings are given on a 0–3 point scale (no/slight/moderate/marked abnormality). The psychometric properties of the Heidelberg Scale are well established in previous studies [2, 31].

The neuropsychological test battery included the Mini Mental State Examination (MMSE) as cognitive screening instrument [32], the subtests logical memory I (immediate recall) and II (delayed recall) from the Wechsler Memory Scale-Revised [WMS-R, 33] for the assessment of verbal memory, the digit span forward and backward tasks from the WMS-R [33] for evaluation of short-term and working memory, Trail Making Test version A and B [TMT, 34] for testing psychomotor speed and cognitive flexibility, respectively, and the verbal fluency task “Animal Category “ from the CERAD (Consortium to Establish a Registry for Alzheimer’s Disease) battery [35].

At T2 diagnoses, psychopathological symptoms, NSS and neuropsychological performance were reassessed using the indicated instruments. The interval amounted to a mean of 7.38 years (± 0.92, range 5–9 years).

### Statistical analyses

Statistical analyses were performed with SPSS version 23 (IBM SPSS Statistics) and α level of 0.05 was applied for all statistical tests.

Dependent *t* tests were used to calculate changes over time in psychopathology, NSS and neurocognition. In case of TMT B, we used McNemar test instead.

Bivariate correlations (Pearson’s *r*) were calculated to analyze potential associations between NSS scores and age and chlorpromazine (CPZ) equivalents, respectively.

Moreover, NSS scores that had changed significantly at the follow-up evaluation (= dependent variables) were entered in a hierarchical stepwise linear regression analysis to detect possible predictors of NSS changes. Age, years of education and duration of illness at T1 were introduced in the model as covariates (method: enter). As predictors, we used those clinical variables at T1 that changed significantly or with trend-level significance over time.

## Results

### Psychopathological characteristics and neuropsychology

As summarized in Table 2, none of the psychopathological characteristics changed significantly during the follow-up period. This was also the case for the average dose of CPZ equivalents.

**Table 2:** Baseline and follow-up psychopathological characteristics

	T1	T2	*t*	*df*	*p*
	Mean (SD)	Mean (SD)			
SAPS Sum Score	15.43 (16.14)	12.62 (12.46)	0.826	20	0.418
SANS Sum Score	25.14 (15.84)	22.52 (15.46)	0.553	20	0.587
AES Sum Score	27.20 (10.49)	26.25 (11.42)	0.508	19	0.617
BPRS Sum score	20.05 (11.19)	15.86 (6.96)	1.456	20	0.161
BPRS Anxiety/depression	7.19 (5.08)	5.57 (3.84)	1.354	20	0.191
BPRS Anergia	5.43 (4.98)	5.10 (3.53)	0.250	20	0.805
BPRS Thought disturbance	4.24 (4.12)	3.43 (3.78)	0.795	20	0.436
BPRS Activity	1.71 (2.05)	0.91 (1.34)	1.602	20	0.125
BPRS Hostility/suspiciousness	1.48 (2.23)	0.86 (1.35)	1.069	20	0.298
Chlorpromazine equivalents, mg	459.62 (274.65)	562.88 (292.44)	− 1.526	20	0.143

*AES* Apathy evaluation scale, *BPRS* Brief psychiatric rating scale, *SANS* Scale for the assessment of negative symptoms, *SAPS* Scale for the assessment of positive symptoms

Patients’ cognitive performance declined significantly over time (see Table 3); this was the case for general cognitive abilities (MMSE, *p* < 0.03), verbal memory (logical memory I and II, *p* < 0.05), verbal fluency (*p* = 0.05) and, trend-level significance only, for psychomotor speed (TMT A, *p* = 0.055). However, three patients could not perform this test at T2. In case of cognitive flexibility (TMT B) about 60% of the patients were not able anymore to perform this test at follow-up assessment. McNemar test demonstrated a statistically significant difference in the proportion of performers of TMT B at T1 and T2 (*p* < 0.001).

**Table 3:** Baseline and follow-up neuropsychological parameters

	T1	T2	*t*	*df*	*p*
	Mean (SD)	Mean (SD)			
Mini Mental State Examination	26.19 (3.88)	24.14 (4.45)	2.495	20	**0.021**
Logical memory I	14.57 (8.18)	11.14 (8.22)	2.498	20	**0.021**
Logical memory II	10.19 (7.05)	7.33 (8.42)	2.107	20	**0.048**
Digit span forward	5.90 (1.71)	5.85 (1.60)	0.160	19	0.874
Digit span backward	4.95 (2.09)	4.20 (2.44)	1.543	19	0.139
Trail making test A	55.28 (23.72)	71.67 (38.01)	− 2.061	17	0.055
Trail making test B	183.52 (68.54)	^a^			**0.000**^b^
Verbal fluency	17.52 (5.29)	15.43 (6.40)	2.071	20	**0.051**

Bold values indicate (*p* ≤ 0.05)

^a^12 patients (= 57.1%) were not able to perform this test at T2

^b^McNemar test (2 sided)

### Neurological soft signs

**Fig. 1:**
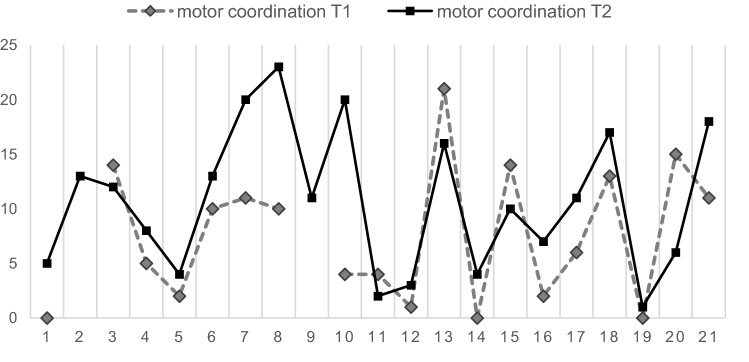
Change of NSS over time (T1, T2) in each individual subject (0–21), NSS subscale “motor coordination”

**Fig. 2:**
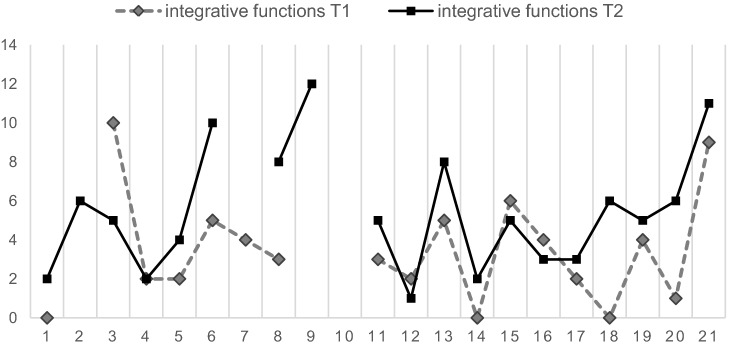
Change of NSS over time (T1, T2) in each individual subject (0–21), NSS subscale “integrative functions”

While patients’ mean initial NSS total score did not change significantly (*p* > 0.08) over time, the scores of the subscales “motor coordination” and “integrative functions” increased significantly (*p* < 0.05) during the follow-up period (Table 4; Figs. 1, 2).

**Table 4:** Baseline and follow-up NSS scores

	T1	T2	t	*df*	p
	Mean (SD)	Mean (SD)			
NSS total score	18.50 (12.47)	24.31 (16.51)	− 1.865	15	0.082
Motor coordination	7.53 (6.21)	10.53 (6.86)	− 2.179	18	**0.043**
Integrative functions	3.41 (2.92)	5.06 (2.88)	− 2.441	16	**0.027**
Complex motor tasks	3.11 (2.51)	3.63 (3.08)	− 0.826	18	0.419
Right/left and spatial orientation	4.25 (3.47)	5.00 (4.93)	− 0.812	15	0.430
Hard signs	1.32 (1.64)	2.68 (2.45)	− 1.728	18	0.101

Bold values indicate (*p* ≤ 0.05)

The calculation of Pearson’s correlation coefficients between NSS total scores and age or CPZ equivalents, respectively, yielded no significant results for T1 (*p* > 0.10), and the same applied for T2 (*p* > 0.06).

To identify predictors of NSS increase, we calculated regression analyses for the subscales “motor coordination” and “integrative functions”, which changed significantly over time. As predictor variables, we used raw scores of the tests MMSE, logical memory I and II, verbal fluency, TMT A and B at T1. These analyses identified verbal fluency and TMT B performance at first examination to be significant predictors for NSS changes, while age, education and duration of illness did not contribute significantly to the models (Table 5).

**Table 5:** Results of regression analyses

	Variable	Standardized Beta	*T*	*p*	*R*^2^	*F*_[df]_	*R*^2^ change	Sign. F change
NSS subscore “motor coordination”								
Model 1					0.170	F[3, 17] = 2.368	0.295	2.368
	Age	0.492	1.768	0.095				
	Education	− 0.279	− 1.314	0.206				
	Duration of illness	0.059	0.219	0.829				
Model 2					0.342	**F**[1, 16] = 3.598*	**0.179**	5.434*
	Age	0.430	1.725	0.104				
	Education	− 0.132	− 0.660	0.518				
	Duration of illness	0.286	1.100	0.288				
	Verbal fluency	− 0.486	− 2.331	0.033				
NSS subscore “integrative functions”								
Model 1					0.160	F[3, 15] = 2.146	0.300	2.146
	Age	0.308	0.935	0.364				
	Education	− 0.297	− 1.254	0.229				
	Duration of illness	0.250	0.793	0.440				
Model 2					0.486	**F**[1, 14] = 5.248**	**0.300**	10.486**
	Age	− 0.108	− 0.376	0.713				
	Education	0.032	0.153	0.880				
	Duration of illness	0.358	1.438	0.173				
	Trail making Test B	0.671	3.238	0.006				

*p ≤ 0.05, **p ≤ 0.01

## Discussion

This study aimed at investigating the course of NSS and cognition in patients with chronic schizophrenia. Two main findings emerged: (1) Scores of the NSS subscales “motor coordination” and “integrative functions” increased significantly during the follow-up interval of 7 years. (2) General cognition, verbal memory as well as executive functions declined significantly over time, while psychopathological symptoms remained rather stable. In addition, executive functions at baseline were the strongest predictors for NSS changes.

Our findings of increasing NSS subscores in middle-aged patients with chronic schizophrenia are well in line with a recent review of the literature [6] that supports a worsening of NSS over time in chronic schizophrenia; an effect which was already demonstrated in a previous meta-analysis [5]. However, older patients with a chronic course of the disorder were only scarcely investigated: a recent cross-sectional study of our own group [19] with a chronically ill patient sample divided into three age groups (mean duration of illness: 3.2, 17.3, 35.6 years) yielded a significant effect of age on NSS, with increasing NSS scores in both, patients and healthy controls with increasing age. This effect was more pronounced in patients than controls, thus indicating that the increase of NSS in chronic schizophrenia exceeded age-associated changes. In contrast, Smith et al. [36] concluded that NSS (Neurological Evaluation Scale/NES) may be a rather stable, trait-like phenomenon in 37 chronically hospitalized patients with schizophrenia (mean duration of illness 22 ± 9 years) which were evaluated repeatedly over a 5-year period.

However, Chen and colleagues [37] examined NSS in 43 stable chronic schizophrenic patients (mean duration of illness 23.5 ± 8.5 years) twice over a 3-year period. Scores on NSS subscales (Cambridge Neurological Inventory/CNI) ‘motor coordination’, ‘sensory integration’ and ‘disinhibition’ increased significantly over time, while symptoms and medication remained largely unchanged as well as the subscales ‘pyramidal’, ‘extrapyramidal’, ‘dyskinesia’ and ‘catatonia’ signs. This NSS increase seemed to be unrelated to age and illness duration, symptoms or medication. These results were interpreted as much as the authors suggested that NSS could be a marker being sensitive to a possible late deterioration process in the course of schizophrenia.

Moreover, Chan et al. [38] recently described NSS as robust biomarkers for the discrimination between individuals in different stages of the schizophrenia spectrum from healthy controls, after having examined NSS in patients with first-episode schizophrenia, individuals with ultra-high risk for psychosis, subjects with schizotypy and healthy controls (*N* = 39 in each group). In addition, it has been shown that certain sub-items of NSS (CNI: motor coordination and total score) and neurocognitive performance (MATRICS Consensus Cognitive Battery: information processing speed, reasoning skill, verbal learning and attention) might be endophenotypes of schizophrenia: remitted patients with schizophrenia, their first-degree unaffected relatives and healthy controls (*N* = 86 in each group) could most accurately (71.2%) be classified with these sub-items working as a composite endophenotype [39]. Taken together, these findings imply that NSS (in combination with neurocognition) may be used as markers for an early identification of both, high-risk individuals and those patients with a rather unfavorable, chronic course.

An accelerated deterioration of executive functions (cognitive flexibility) in elderly patients with chronic schizophrenia has been recently confirmed in an own cross-sectional design [12], as previous studies already described a worsening of executive functions, processing speed and verbal learning cross-sectionally [40–42]. These findings were extended in the present study which identified executive dysfunction as a significant predictor of NSS increase over time in chronic schizophrenia.

In contrast, Heaton et al. [43], who investigated 142 patients with chronic schizophrenia (mean duration of illness: 19 ± 14 years) reported neuropsychological impairments in ambulatory persons with schizophrenia to remain rather stable over 5 years. However, authors emphasized that these results may not be generalizable to institutionalized poor-outcome patients, which were not represented by their study. Similarly, Savla et al. [44] found cognitive impairments to be stable over time (on average: 15 months) among 143 outpatients with schizophrenia (mean age: 53 ± 9 years), though, the follow-up interval in this trial was much shorter than in the present study.

According to our results, deteriorating NSS in the longitudinal course of chronic schizophrenia go along with decreasing performance in executive functions and verbal memory. This effect may be attributed to premature brain aging following the concept of accelerated aging in schizophrenia [45], which parallels Kraepelin’s original concept of dementia praecox [46]. Severe concomitant diseases such type II diabetes mellitus or hypertension may provide an additional explanation, since the latter are associated with cognitive impairments in an otherwise healthy population [47, 48].

Besides NSS and cognitive impairments psychopathological symptoms are affected by age and the aging process. While the severity of positive symptoms seems to diminish slightly in later life [for overview see: 16, 17], negative symptoms are generally considered to increase in the older, chronically ill; however, this dissociation was only partially confirmed in classical studies, which also found a “second, positive knick” even decades after manifestation of the disease instead [49–51]. In our sample no significant psychopathological changes were found longitudinally. Similarly, three symptomatological dimensions (negative, disorganized, paranoid) could be extracted by factor analysis in a group of 131 chronic schizophrenic patients (mean duration of illness: 40 ± 9 years). These dimensions correspond to those found in younger chronic patients and were independent of the severity of cognitive deficits [52]. Consistent with our results, a recent meta-analysis and review of Heilbronner and colleagues [53] described a rather stable psychopathological profile with worsening of cognition in later life associated with neurodegeneration.

In general, NSS in younger patient samples are structurally associated with morphological alterations of pre- and post-central gyri, premotor area, cerebellum, middle and inferior frontal gyri, thalamus and basal ganglia, temporal and lingual gyri, inferior parietal lobule, insula, precuneus and occipital gyrus [for review see: 54, 55].

In addition, Kong et al. [7] found the dichotomisation into patients with decreasing and patients with persistent NSS levels supported by different underlying cerebral changes over time, which were more pronounced in those prone to a rather unfavorable course. These results were extended by findings of a more recent publication about the associations of GM with NSS scores in 49 patients with chronic schizophrenia (mean duration of illness of 20.3 ± 14.0 years). We have identified the lingual, parahippocampal, superior temporal, inferior and middle frontal gyri, thalamus and cerebellum as important sites of NSS in chronic schizophrenia [56]. Thus, corresponding to our own longitudinal results [7] we confirmed in our recent study significant negative correlations between GM in frontal and cerebellar regions and NSS scores [56]. These findings support our hypothesis of associations between increasing NSS in patients with chronic schizophrenia, as shown in the present study, and progressive cerebral changes. Taken together these results also correspond with the assumption of a disrupted cortico–cerebellar–thalamic–cortical circuit in schizophrenia, conceptualized as model of “cognitive dysmetria” [57].

Sample size and concomitant diseases have to be considered as potential confounders. However, the follow-up assessment of older patients with a chronic course of the disease was difficult due to the institutionalization of one third of the patients in nursing homes at the second evaluation [see also: 58]. Given the severity of the disease not all patients were able to complete the entire examination. However, a physically healthy sample of middle-aged patients with chronic schizophrenia would be far away from being representative. Indeed, it has been shown—as in our sample—that individuals with schizophrenia have high levels of medical comorbidity and cardiovascular risk factors with high rates of metabolic syndrome of 32.3% in medicated patients, while the strongest influence on the rate of metabolic syndrome is illness duration [59–61]. In older patients with a mean age of 50 years or higher metabolic syndrome was present in 39.2%, in contrast to approximately 10% when only first-episode patients were considered [59, 62]. Thus, a poor physical health status may contribute to both, increasing NSS scores and cognitive deficits [22], together with the corresponding cerebral changes, as these parameters seem to worsen over time despite a rather stable psychopathology.

These results underline the need for specific interventions to decrease the respective risk factors, which has been shown to be effective [63]. Also consequent monitoring and treatment of cardiac and metabolic problems [64–67] are necessary, given the high percentage of somatic comorbidities especially in older patients with schizophrenia [68].

## Footnotes

1 In case of NSS-items we had the following number of missing data: NSS total score (T1:3/T2:3), motor coordination (T1:2/T2:0), integrative functions (T1:3/T2:2), complex motor tasks (T1:2/T2:0), right/left and spatial orientation (T1:2/T2:3), hard signs (T1:2/T2:0). If one item was missing, the respective subscore and the sumscore were not calculated.

2 Motor coordination: Ozeretski’s test, diadochokinesis, pronation/supination, finger-to-thumb opposition, speech and articulation.

3 Integrative functions: gait, tandem walking, two-point discrimination.

4 Complex motor tasks: finger-to-nose test, fist-edge-palm test.

5 Right/left and spatial orientation: right/left orientation, graphesthesia, face-hand test, stereognosis.

6 Hard signs: arm-holding test, mirror movements.
